# RP-HPLC and UV Spectrophotometric Analysis of Paracetamol, Ibuprofen, and Caffeine in Solid Pharmaceutical Dosage Forms by Derivative, Fourier, and Wavelet Transforms: A Comparison Study

**DOI:** 10.1155/2020/8107571

**Published:** 2020-02-08

**Authors:** Hoang Vu Dang, Huong Truong Thi Thu, Ly Dong Thi Ha, Huong Nguyen Mai

**Affiliations:** Department of Analytical Chemistry and Toxicology, Hanoi University of Pharmacy, 13–15 Le Thanh Tong, Hanoi, Vietnam

## Abstract

Different signal-transforming algorithms were applied for UV spectrophotometric analysis of paracetamol, ibuprofen, and caffeine in ternary mixtures. Phosphate buffer pH 7.2 was used as the spectrophotometric solvent. Severe overlapping spectra could be resolved into individual bands in the range of wavelengths 200–300 nm by using Savitzky–Golay smoothing and differentiation, trigonometric Fourier series, and mother wavelet functions (i.e., sym6, haar, coif3, and mexh). To optimize spectral recoveries, the concentration of various types of divisors (single, double, and successive) was tested. The developed spectrophotometric methods showed linearity over the ranges 20–40 mg/L for paracetamol, 12–32 mg/L for ibuprofen, and 1–3.5 mg/L for caffeine (*R*^2^ > 0.990). They could be successfully applied to the assay and dissolution test of paracetamol, ibuprofen, and caffeine in their combined tablets and capsules, with accuracy (99.1–101.5% recovery) and precision (RSD < 2%). For comparison, an isocratic RP-HPLC analysis was also developed and validated on an Agilent ZORBAX Eclipse XDB–C18 (150 × 4.6 mm, 5 *µ*m) at an ambient temperature. A mixture of methanol : phosphate buffer 0.01 M pH 3 (30 : 70 v/v) was used as the mobile phase delivered at 2 mL/min, and the effluent was monitored at 225 nm. It was shown that spectrophotometric data were statistically comparable to HPLC (*p* > 0.05), suggesting possible interchange between UV spectrophotometric and HPLC methods for routine analysis of paracetamol, ibuprofen, and caffeine in their solid pharmaceutical dosage forms.

## 1. Introduction

Combined pharmaceutical dosage forms have been more and more manufactured and used for better treatment outcome. There was considerable clinical evidence that a multicomponent therapy (like combined analgesics) is more efficient than monocomponent therapies because it can expand the array of therapeutic options, facilitate the completeness of therapeutic effect, and permit the doctors (and, in self-medication with over-the-counter (OTC) medications, the patients themselves) to personalize any treatment based on the patient's specific needs [1].

**Figure 1:**
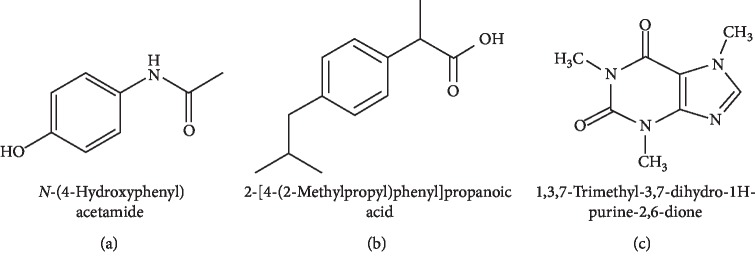
Chemical structures and IUPAC names of paracetamol (a), ibuprofen, (b) and caffeine (c).

Paracetamol (a.k.a. acetaminophen, Figure 1(a)) was popularly used to combat fever in the 1950s and now becomes the antipyretic and analgesic of first choice in most countries [2]. It is thought to increase human pain threshold by reducing the production of prostaglandins in the brain and spinal cord. Despite of having few adverse effects and little interaction with other pharmaceutical agents, this drug exhibits very little anti-inflammatory activity probably due to its inability to suppress COX in the presence of the elevated cellular peroxides found in inflamed cells [3]. To enhance the painkilling effect, paracetamol can be orally taken in combination with ibuprofen (a nonsteroidal anti-inflammatory drug, Figure 1(b)) and caffeine (a mild neurostimulant that produces cerebral vasoconstriction by antagonizing adenosine receptors and helps reduce fatigue, Figure 1(c)) [4].

To analyze paracetamol in multicomponent commercial products, chromatographic techniques (reversed-phase high-performance liquid chromatography (RP-HPLC) and capillary electrophoresis (CE)) connected with UV detection have been numerously investigated since 1990s [5–10] as well as regulated in many pharmacopoeias (e.g., United State pharmacopoeia and British pharmacopoeia [11, 12]). These methods may be eco-unfriendly, expensive, and tedious due to complicated instruments and organic solvents used. Bearing this in mind, UV spectrophotometry coupled with chemometric tools was also studied for simultaneous determination of active compounds in binary and ternary mixtures containing paracetamol without the assistance of any pretreatment process by chemical and/or separation means [13–29].

In analytical chemistry, signal transforms (derivative, Fourier, and wavelet) demonstrated to be an encouraging means to deconvolve UV overlapping spectra of ternary and quaternary mixtures [30–33]. Based on this signal-processing approach, the present study aims at developing analytical methods based on signal-transforming UV ratio spectra for the simultaneous determination of paracetamol, ibuprofen, and caffeine in coformulated ternary mixtures, using RP-HPLC as a reference method. It specifically assesses the applicability of developed UV spectrophotometric methods for both assay and dissolution test of paracetamol, ibuprofen, and caffeine in their combined solid pharmaceutical dosage forms.

## 2. Experimental Setup

### 2.1. Apparatus and Software

A UNICAM UV 300 double beam spectrophotometer (Thermo Spectronic, USA) was used with a 1.5 nm fixed slit width and equipped with 1 cm quartz cuvettes. The absorption spectra were registered between 190 and 325 nm, using phosphate buffer pH 7.2 as blank. The spectrophotometer operated at the data interval fixed at Δλ = 0.1 nm, and the scan speed varied (i.e., 30–120 nm/min) to boost the spectral signal-to-noise ratio without the prolonged scan period. For derivative transform, the ratio spectra were subjected to Savitzky–Golay differentiation and smoothing (Thermo Spectronic VISION32 software). For Fourier and wavelet transforms, the data treatment was performed by using Microsoft Excel and Wavelet Toolbox, MATLAB R2015a software (The MathWorks, Inc. USA), respectively.

RP-HPLC analysis was carried out on an Agilent 1200 Infinity Series Diode-Array-Detector liquid chromatograph (Agilent Technologies, Inc. USA) installed with a ZORBAX Eclipse XDB-C18 (150 × 4.6 mm; 5 *µ*m) column. Before injection into the chromatograph, all solutions were filtered through a 0.45 *μ*m membrane. The HPLC running buffer was also subjected to a microfiltration (0.45 *μ*m MF-Millipore™ membrane) and underwent ultrasonic degassing before use. Chromatographic variables were investigated, i.e., different mobile phase compositions (organic modifier: aqueous buffer ratios (20 : 80–40 : 60 v/v)), flow rates (1.0–2.0 mL/min), and column temperatures (20–40 C).

Dissolution test was performed at 37 C by using the dissolution medium (phosphate buffer pH 7.2) on an Erweka DT 626 (Erweka GmbH, Germany) dissolutor (six vessels containing 900 mL of the dissolution medium) with a paddle apparatus set at 150 rpm.

### 2.2. Reagents and Standard Solutions

Paracetamol (PA), caffeine (CA), and ibuprofen (IB) primary reference standards were provided from the National Institute of Drug Quality Control (Vietnam). All chemicals used were of analytical grade.

The spectrophotometric solvent was made up of 250 mL of potassium dihydrophosphate, 0.2 M and 175 mL of sodium hydroxide, and 0.2 M in a final volume of 1 L with deionized double-distilled water. The preparation of a set of standard solutions was accomplished in 100-mL volumetric flasks by using stock solutions freshly prepared in the same solvent (PA 500 mg/L, CA 200 mg/L, and IB 500 mg/L).

### 2.3. Sample Solutions

Three Vietnamese formulations were purchased in the local market (i.e., Glotasic (PA 325 mg + IB 200 mg + CA 25 mg per tablet), Glomed Pharmaceutical Co., Inc., Bidi-Ipalvic (PA 300 mg + IB 200 mg + CA 20 mg per capsule), BinhDinh Pharmaceutical and Medical Equipment Joint Stock company, Ibu-Acetalvic (PA 300 mg + IB 200 mg + CA 20 mg per capsule), and Vidipha Central Pharmaceutical Joint Stock company). For each dosage form, the powder of 20 crushed tablets or the content of 20 capsules was thoroughly mixed.

The test solution, ca. PA 32.5 (30) mg/L + IB 20 mg/L + CA 2.5 (2) mg/L, was obtained in a 100-mL volumetric flask by ultrasonic dissolution and appropriate dilution of an accurate quantity equivalent to one tenth of a tablet or capsule.

Dissolution samples were collected at predetermined time intervals within a time period of 1 hour, appropriately diluted with the spectrophotometric solvent (2–10 times), and filtered before spectral measurement.

## 3. Results and Discussion

### 3.1. Method Development

#### 3.1.1. Spectrophotometric Methods

Theoretically speaking, the three drug molecules under study (PA, IB, and CA) can absorb light of certain wavelength ≥ 200 nm, allowing electrons in the valence band to jump from the ground state to excited state because they have both chromophores (benzene ring, carbonyl group, carboxyl group, and heterocyclic nitrogen ring) and auxochromes (hydroxyl group, amide group, and alkyl group).

**Figure 2:**
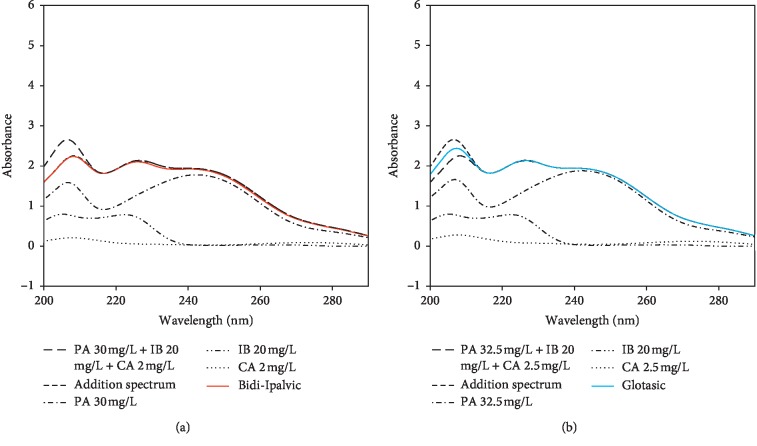
Spectra of 32.5 (30) mg/L PA, 20 mg/L IB, 2.5 (2) mg/L CA, and their corresponding ternary mixture, additivity, and Bidi-Ipalvic (a) or Glotasic (b).

It is truly justified by data displayed in Figures 2(a) and 2(b) (i.e., the zero-order UV spectra being subjected to Savitzky–Golay smoothing filter (3^rd^ order polynomial and 125 convolution coefficients)). Suppose that the content of all drugs complies with label claim, the existence of inactive substances in their solid pharmaceutical dosage forms does not modify considerably the spectral characteristics of ternary mixtures of PA 32.5 (30) mg/L + IB 20 mg/L + CA 2.5 (2) mg/L. However, a significant spectral overlap is seen in the range 200–300 nm for PA, IB, and CA. As PA's spectral absorption dominated over the wavelength region studied, the coassay of all drugs in their combined mixtures is inhibited by traditional UV spectrophotometry, especially for IB and CA. It is worth mentioning that phosphate buffer pH 7.2 was chosen as the spectrophotometric solvent herein because it could well dissolve all drugs in the range of concentrations investigated and was also suggested for the dissolution test of IB tablets [11].

Unlike previously published data (i.e., using multivariate concentration determination: partial least-squares regression (PLS), genetic algorithm coupled with PLS (GA-PLS), and principal component-artificial neural network (PC-ANN) [21], a fully questionable determination of zero-crossing points with no divisor [34, 35]), in this study, different algorithms (i.e., derivative, Fourier, and wavelet transforms) were employed to separate severe overlapping spectra of PA-IB-CA ternary mixtures into individual bands for quantification. In principle, these transforms could be done with UV ratio spectra using different types of divisor: single [33], double [36], and successive [37] as once proposed. With a single divisor, detecting crossing or zero-crossing points is prerequisite. In contrast, the appropriateness of other divisors counts on locating signal coincidence at a region or for the transformed spectra of a compound and its corresponding mixture. To obtain the highest spectral recovery, the choice of relevant divisor concentrations and signal-transforming functions for transformed ratio spectra must be taken into consideration. Considering the virtue of clarity, the transformed ratio spectra were distinctly presented for PA 32.5 (30) mg/L, IB 20 mg/L, CA 2 mg/L, and their corresponding mixture to point out the working wavelengths for double and successive divisor ratio spectrophotometric methods.

*(1) Derivative Transform*. The differentiation and smoothing of UV spectra were done by using the Savitzky–Golay algorithm [38], optimally fitting a data subset of 2*n* + 1 measurement points to a polynomial in the least-squares sense.

**Figure 3:**
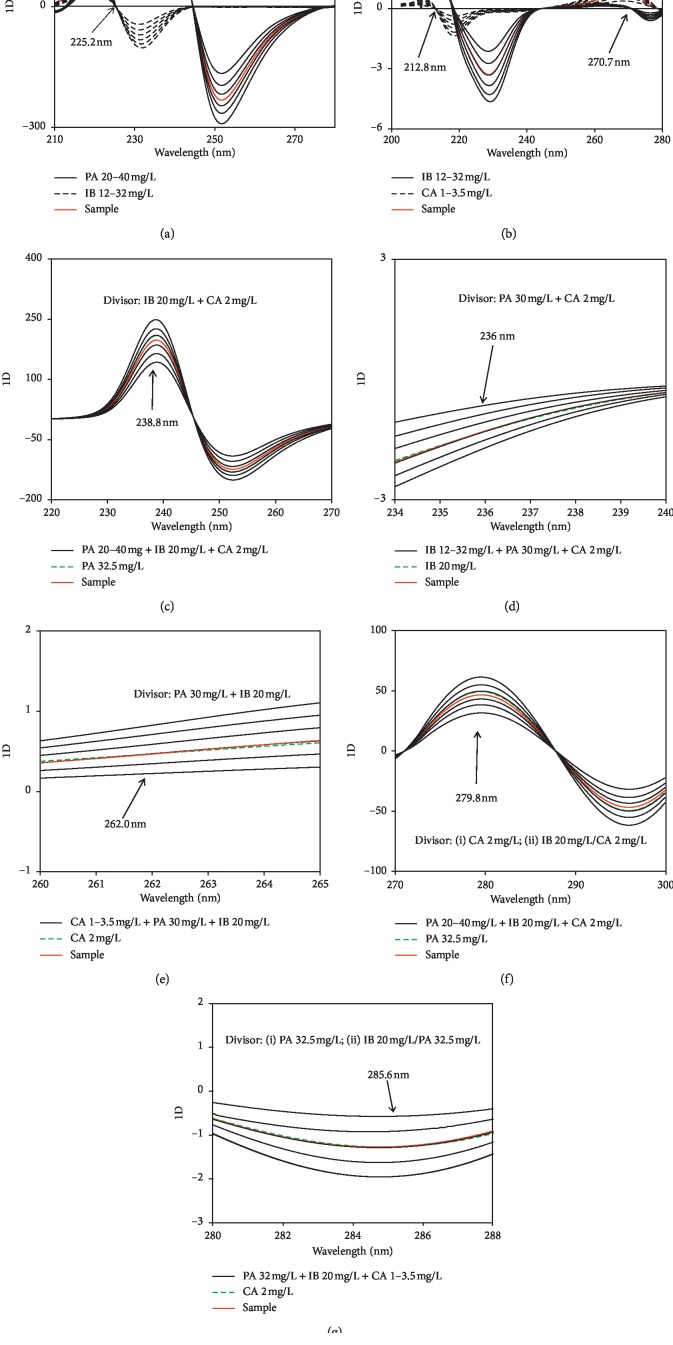
Using derivative transform for determination of PA, IB, and CA: single divisors (a, b), double divisors (c–e), and successive divisors (f, g).

Using the derivative transform with single divisor, the ratio spectra were obtained by dividing PA and IB spectra by a spectrum of CA 2 mg/L. These ratio spectra were computed for their first-order derivative (5^th^ order polynomial and 125 convolution coefficients) and subsequently smoothed (3^rd^ order polynomial and 125 convolution coefficients) by using Savitzky–Golay filters (Figure 3(a)). In the PA-IB-CA mixture, the concentration of PA was figured out by measuring the derivative amplitude at 225.2 nm (IB zero-crossing point). Similarly, the concentrations of IB and CA were achieved with the working wavelengths of 212.8 nm (CA zero-crossing point) and 270.7 nm (IB zero-crossing point), respectively, when IB and CA spectra were being divided by a spectrum of PA 30 mg/L (Figure 3(b)).

Under other conditions, the assay of PA could be done with double divisor (i.e., the ratio spectra were obtained by dividing the UV spectra of PA-IB-CA ternary mixtures by the spectrum of an IB-CA binary mixture). In contrast to Dinç's proposal [33, 36] specifying that a double divisor contains approximately equimolar concentrations of each component, the binary mixture of IB 20 mg/L + CA 2 mg/L was established as an acceptable double divisor to determine PA at 238.8 nm. It is a peak wavelength of the first-order derivative of the ratio spectra in the spectral region coexisted for PA 32.5 mg/L and its ternary mixture of PA 32.5 mg/L + IB 20 mg/L + CA 2 mg/L (Figure 3(c)). In a similar manner, the binary mixtures of PA 30 mg/L + CA 2 mg/L CA and PA 30 mg/L + IB 20 mg/L were used as double divisors to determine IB and CA at 236.0 and 262.0 nm, respectively (Figures 3(d) and 3(e)).

Moreover, PA and CA could be also quantified in their ternary mixtures with successive divisors. For example, the first ratio spectra were obtained when the spectra of PA containing ternary mixtures was divided by a spectrum of CA 2 mg/L (the first divisor). These ratio spectra were first-order differentiated and subsequently divided by the second divisor (the first-order derivative of the ratio spectrum acquired by dividing IB 20 mg/L by CA 2 mg/L). Thereafter, the second ratio spectra were subjected to first-order differential computation and again smoothing to expose the working wavelength of 279.8 nm (Figure 3(f)). In the same way, CA was determined at 285.6 nm with successive divisors ((i) PA 32.5 mg/L; (ii) IB 20 mg/L/PA 32.5 mg/L) (Figure 3(g)), whereas no working wavelength was found for IB determination with successive divisors.

It is obviously seen that the amplitude of derivative signals in our study is much higher than that reported in others [29, 34, 35].

**Figure 4:**
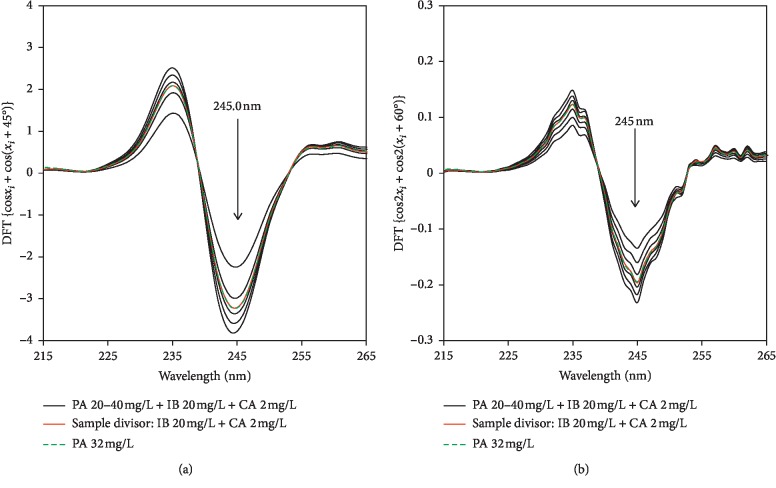
Using discrete Fourier transform for determination of PA: double divisor (a, b).

*(2) Discrete Fourier Transform*. Using the discrete Fourier transform, the UV spectra at a set of (*n* + 1) equally spaced wavelengths could be expanded as the sum of deterministic continuous trigonometric functions sine and cosine [39]. This approach, however, was only able to help quantify PA in PA-IB-CA ternary mixtures using double divisor with all trigonometric functions readily available; i.e., the spectra in the range 210–300 nm were deconvolved, and a set of Fourier coefficients were calculated by using 6- or 8-point combined trigonometric functions (i.e., cos*x*_*i*_ + cos(*x*_*i*_ + 60 ); cos2*x*_*i*_ + cos2 (*x*_*i*_ + 60 ); sin*x*_*i*_–sin (*x*_*i*_ + 60 ); sin2*x*_*i*_–sin 2(*x*_*i*_ + 60 ); cos*x*_*i*_ + cos(*x*_*i*_ + 45 ); cos2*x*_*i*_ + cos2 (*x*_*i*_ + 45 ); sinx_i_–sin (*x*_i_ + 45 ); and sin2*x*_*i*_–sin 2(*x*_*i*_ + 45 )). Figures 4(a) and 4(b) exhibit some representative discrete Fourier-transformed signals of ratio spectra with ∆*λ* = 1 nm (i.e., the highest and smallest signal amplitudes obtained with discrete Fourier transform). It is noted that although the discrete Fourier transform generated lower amplitude signals as compared with corresponding derivative ones, there was no need to perform an additional smoothing step to Fourier-transformed signals as already explained [40].

*(3) Wavelet Transform*. In mathematics, a wavelet transform (WT) means that a signal is decomposed into a set of basic functions that are related to dilation and scaling of a transforming function *ψ*(*t*), so-called the “mother wavelet” [41]. In other words, WT is dependent upon the approval of a single prototype wavelet function for generating the other window functions. With reference to spectral analysis, WT has strongly proved to be one of the most crucial and attractive signal-processing algorithms for noise elimination, background amelioration, differentiation, data smoothing and filtering, data compression, and separation of signals with overlapping spectra, etc. [42]. In particular, WT-based UV spectroscopy has been profitably utilized for the assay of pharmaceuticals in binary and ternary mixtures [43]. This is because the original signal or function could be mathematically expressed in terms of a linear combination of wavelets (i.e., a sum of a series where each term is the product of a constant coefficient and a wavelet), making the wavelet transform of UV spectra feasible.

**Figure 5:**
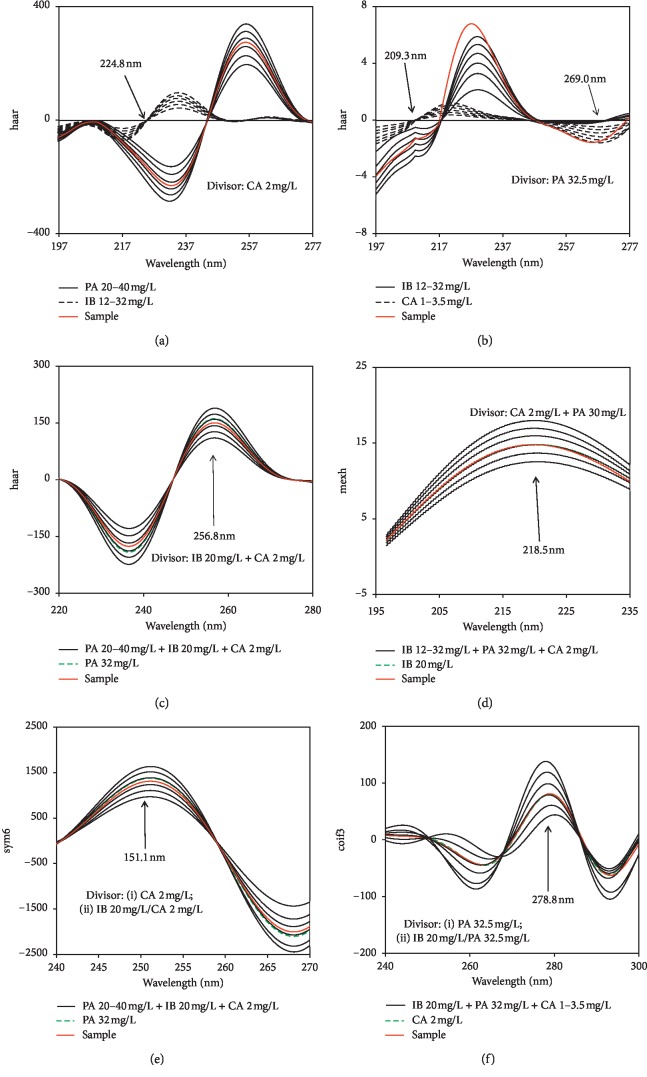
Using wavelet transform for determination of PA, IB, and CA: single divisors (a, b), double divisors (c, d), and successive divisors (e, f).

Using the wavelet transform, the signal decomposition of UV ratio spectra of PA-IB-CA ternary mixtures into individual bands was scrutinized with all MATLAB built-in wavelet families. Sym6, haar, coif3, and mexh were mother wavelets chosen for signal-transforming ratio spectra with single, double, and successive divisors. WTs were optimally performed at the scaling factor, *a* = 256. Figures 5(a)–5(f) display representatively UV ratio spectra resolved by using these mother wavelets. It is obvious that the intensity of transformed signals has been remarkably augmented by WT as compared to derivative transform, especially for IB and CA (minor components in mixtures) transformed ratio spectra.

#### 3.1.2. Reversed-Phase High-Performance Liquid Chromatography Method

As stated in the United States Pharmacopeia [11], chromatographic analysis was applied to the assay of these drugs using the C18 column and UV detection. The mobile phases are regulated to be (i) a mixture of methanol: water: glacial acetic acid (28 : 69 : 3 v/v/v) for PA-CA combined tablets, and (ii) a mixture of acetonitrile and water (containing chloroacetic acid 1% and being pH-adjusted to 3.0 with ammonium hydroxide) (6 : 4 v/v) for IB tablets.

For comparison, the chromatographic determination was investigated to concurrently analyze PA, IB, and CA in their ternary mixtures. The RP-HPLC development was done with a 150 × 4.6 mm, 5 *µ*m ZORBAX Eclipse XDB–C18 column installed on the system.

This column, containing ultra-high purity Zorbax Rx-SIL porous silica support ( ≥ 99.995% SiO_2_), could be resistant to mobile phases of intermediate and higher pH values, thanks to (i) a dense monolayer of dimethyl-*n*-octadecylsilane stationary phase chemically bonded (eXtra-Dense Bonding (XDB) technologies) and (ii) double endcapping. This densely covered, deactivated column packing is exclusively beneficial for HPLC analysis of acidic, basic, and other highly polar analytes (e.g., CA).

To attain a good RP-HPLC performance, chromatographic conditions (i.e., flow rate, injection volume, organic modifier type and ratio, buffer pH, and concentration) were scrutinized. In RP-HPLC, the hydrophobicity of an analyte molecule (often expressed as log *P*) is usually the primary indicator of its retentivity. In literature, log *P* values are 0.46 [44], –0.07 [45], and 3.97 [46] for PA, CA, and IB, respectively. Provided that the higher the value of log *P* the more hydrophobic the molecule, it reasonably agrees with the observation that IB was the last eluting compound. In contrast, the fact that PA was eluted out before CA and could be attributable to some limitation of log *P* value (only describing the partition coefficient of neutral or unionized drug molecules).

With regard to the chemical structure, CA is both a weak acid and a weak base with pKa values of 14.0 (tertiary amine) and 0.7 (caffeine cation) [47, 48]. As the degree of ionization also dictates the analyte hydrophobicity (ionic analytes are much less hydrophobic) and possibly shows secondary interactions with the column (free silanol groups), the polarity of the mobile phase was enhanced by addition of the 10–50 mM phosphate buffer over the pH range 3–7. It makes CA predominantly protonated species, and CA is probably less polar than PA in this chemical form.

It is reported that methanol, acetonitrile, and/or combinations of these two modifiers are the most commonly used organic solvents for the RP-HPLC method development purposes. Nevertheless, several PA-CA coelutions were observed when acetonitrile was used (data not shown). When methanol was used, different selectivity was produced and all drugs were fully resolved.

The following condition was selected as optimal for RP-HPLC analysis. PA, IB, and CA were separated by isocratic liquid chromatography with a flow rate of 2.0 mL/min at an ambient temperature of 25 C. The eluent was composed of methanol : phosphate buffer (KH_2_PO_4_ 0.01 M pH 3 and phosphoric acid used for pH adjustment) (30 : 70 v/v). Sample injection volume was 20 *μ*L, and PDA detector was set at 225.0 nm.

**Figure 6:**
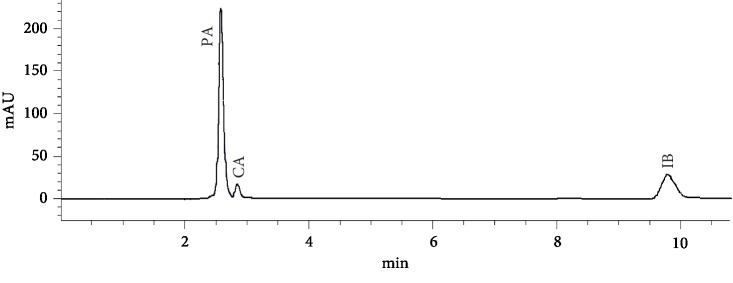
Typical RP-HPLC chromatogram of a ternary mixture containing PA 32.5 mg/L, IB 20 mg/L, and CA 2 mg/L.

Under this chromatographic condition, PA, CA, and IB were eluted at 2.58, 2.85, and 9.81 min, respectively (Figure 6). The suitability of our HPLC method could be apparently assured for all drugs with respect to basic chromatographic parameters describing a resolution between two adjacent peaks (*R*_s_ > 1.5), peak asymmetry factor (*A*_*s*_ = 0.8–0.9), and column efficiency (theoretical plate number, *N* > 5500/15 cm).

### 3.2. Method Validation and Application

The validity of all the developed methods was assessed in terms of precision, accuracy, and linearity according to ICH guidelines [49]. The repeatability (within-run precision) was evaluated by RSD for six replicate determinations of the same sample. The accuracy was evaluated by % recovery using the standard addition technique (known amounts of three analytes equal to 20% of their label claim were directly added to the aliquots of analyzed tablets and capsules). RP-HPLC analysis showed to be fairly precise (RSD values < 2.0 %) and accurate (percent recovery ranged 99.5–101.1%). The calibration curves demonstrated a good linear relationship (*R*^2^ > 0.990) over the concentration ranges of PA (20–40 mg/L), IB (12–32 mg/L), and CA (1–3.5 mg/L). In a similar manner, these ranges of linearity were also spectrophotometrically established (Table 1). For Fourier-transformed ratio spectra, exceptionally, the content of PA was determined by using the plots of signal amplitude versus natural logarithm of concentration. Indication of good precision (evaluated by RSD values < 2%) and accuracy (evaluated by recovery percentages 99.1–101.5%) was also found for all spectrophotometric methods.

**Figure 7:**
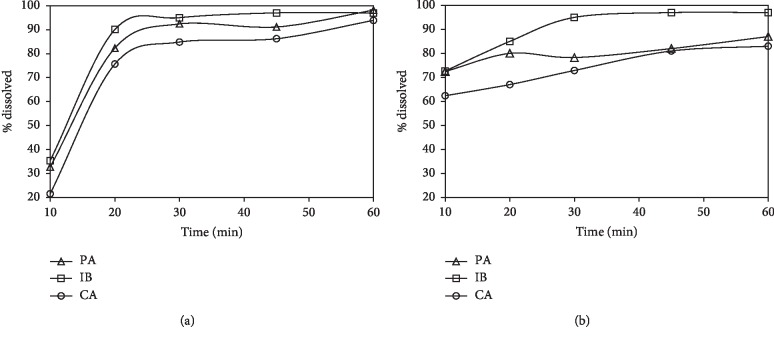
Dissolution profiles of Bidi-Ipalvic capsules (a) and Glotasic tablets (b) determined by using wavelet transform of UV ratio spectra (*n* = 6, RSD < 5%).

The RP-HPLC method was well applied to the simultaneous determination of PA, IB, and CA in their combined tablets and capsules. Moreover, the content of PA, IB, and CA could be successfully coassayed by using derivative-, Fourier-, and wavelet-transformed spectrophotometric methods, implying negligible interfering excipients used in the solid pharmaceutical dosage forms under study (Table 2).  Figure 7 displays the dissolution profiles of PA, IB, and CA, clearly showing that > 80% of the label content released after 30 min for capsules and 45 min for tablets.

Table 3 presents statistical analysis for comparison of spectrophotometric and RP-HPLC assay of PA, IB, and CA in their combined tablets and capsules. At the 95% confidence level, no statistically significant difference was seen in the precision (Bartlett test checking homogeneity of variances of all groups, observed *χ*^2^ value < critical *χ*^2^ value) and accuracy (one-way ANOVA test comparing means of all groups, observed *F* value < critical *F* value) among all the proposed methods.

## 4. Conclusion

UV ratio spectrophotometric methods, which are relied on the first-order derivative and Fourier and wavelet transforms, were developed for the estimation of PA, IB, and CA in ternary mixtures requiring no prior purification and/or separation step. These methods proved to be precise (RSD < 2%) and accurate (in the range of 98–102% recovery). It is clearly observed that wavelet transform outperformed derivative and Fourier transforms with regard to improving the sensitivity of the assay (i.e., signal strength greatly enhanced).

The developed spectrophotometric methods showed statistically comparable precision and accuracy, but more eco-friendly, cost-effective, and time-saving than RP-HPLC. Thus, they could be used for the routine assay and dissolution test of commercially available tablets and capsules simultaneously containing PA, IB, and CA.

## Figures and Tables

**Table 1:** Statistical analysis of the calibration graphs of the proposed spectrophotometric methods for PA (20–40 mg/L), IB (12–32 mg/L), and CA (1–3.5 mg/L) (*n* = 6).

Method divisor (mg/L)	Function	Compound	Wavelength (nm)	*a*	*b*	*S*_*a*_	*S*_*b*_	*S*_*y*·*x*_	*R* ^2^
Derivative transform									
Single divisor									
CA 2	^1^D	PA	225.2^1^	3.2058	5.5814	0.0403	1.2421	0.6763	0.9994
PA 30	^1^D	IB	212.8^1^	0.1072	0.2296	0.0015	0.0357	0.0259	0.9992
PA 30	^1^D	CA	270.7^1^	0.3426	0.0074	0.0042	0.0102	0.0088	0.9994

Double divisor									
IB 20 + CA 2	^1^D	PA	238.8^1^	5.2637	38.076	0.0535	1.6502	0.8985	0.9996
PA 30 + CA 2	^1^D	IB	236.0^1^	−0.0535	0.0019	0.0006	0.0130	0.0095	0.9996
PA 30 + IB 20	^1^D	CA	262.0^1^	0.2512	−0.0319	0.0052	0.0126	0.0110	0.9983

Successive divisor									
CA 2; IB 20/CA 2	^1^D	PA	279.8^1^	1.4573	2.6761	0.0302	0.9307	0.5067	0.9983
CA 2; PA 32.5/CA 2	^1^D	IB	243.2^1^	0.0068	−0.0206	0.0002	0.0045	0.0032	0.9968
PA 32.5; IB 20/PA 32.5	^1^D	CA	285.6^1^	−0.6723	0.1036	0.0076	0.0183	0.0159	0.9995

Fourier transform^*∗*^									
Double divisor									
IB 20 + CA 2	cos*x*_*i*_ + cos (*x*_i_ + 60°)	PA	235.0	0.8681	−1.8316	0.0183	0.0620	0.0106	0.9977
	cos2*x*_*i*_ + cos2 (*x*_*i*_ + 60°)	PA	245.0	−0.1417	0.2900	0.0026	0.0090	0.0015	0.9982
	sin*x*_*i*_ – sin (*x*_*i*_ + 60°)	PA	235.0	−0.5012	1.0574	0.0105	0.0358	0.0061	0.9977
	sin2*x*_*i*_ – sin2(*x*_*i*_ + 60°)	PA	245.0	0.2454	−0.5024	0.0046	0.0156	0.0026	0.9982
	cos*x*_*i*_ + cos(*x*_*i*_ + 45°)	PA	245.0^1^	−2.2482	4.4903	0.0460	0.1556	0.0266	0.9979
	cos2*x*_i_ + cos2(*x*_*i*_ + 45°)	PA	245.0	−0.3837	0.7838	0.0061	0.0207	0.0035	0.9987
	sin*x*_*i*_ – sin (*x*_*i*_ + 45°)	PA	245.0	0.9312	−1.8599	0.0190	0.0644	0.0110	0.9979
	sin2*x*_*i*_ – sin 2(*x*_*i*_ + 45°)	PA	237.0	−0.5673	1.1944	0.0132	0.0448	0.0076	0.9972

Wavelet transform									
Single divisor									
CA 2	sym6	PA	215.8	−2.7258	−21.640	0.5012	1.5803	0.8604	0.9986
	haar	PA	224.8	−5.0086	−16.222	0.0954	2.9443	1.6030	0.9986
	coif	PA	216.6	−2.5529	−18.769	0.0407	1.2567	0.6842	0.9990
	mexh	PA	251.3^1^	15.051	74.527	0.1359	4.1929	2.2828	0.9997

IB 20	sym6	PA	247.6	9.4930	80.257	0.2992	9.2315	5.0261	0.9960
	haar	PA	258.5	9.2566	70.689	0.1533	4.7315	2.5761	0.9989
	coif3	PA	226.0	−5.6236	−47.161	0.1208	3.7285	2.0300	0.9982

PA 32	sym6	IB	219.5	0.1235	−0.0457	0.010	0.0221	0.0161	0.9998
	sym6	CA	227.6^1^	−0.1774	−0.0040	0.0022	0.0052	0.0045	0.9994
	haar	IB	209.3	−0.1016	0.7232	0.0016	0.0371	0.0269	0.9990
	haar	CA	269.0	0.3011	0.0134	0.0046	0.0110	0.0096	0.9991
	coif3	IB	220.3	0.1259	−0.0264	0.0011	0.0243	0.0176	0.9997
	coif3	CA	228.6	−0.1704	−0.0075	0.0018	0.0044	0.0038	0.9995
	mexh	IB	225.3^1^	0.2874	0.847	0.0041	0.0950	0.069	0.9992

Double divisor									
PA 30 + CA 2	sym6	IB	235.9	−0.1158	−0.5166	0.0027	0.0612	0.0444	0.9979
	haar	IB	229.8	0.1606	0.2217	0.0032	0.0744	0.0540	0.9984
	coif3	IB	218.5	0.1617	−1.1876	0.0020	0.0461	0.0335	0.9994
	mexh	IB	220.1^1^	0.2733	9.3074	0.0039	0.0890	0.0646	0.9992

IB 20 + CA 2	sym6	PA	298.5	2.4206	8.2337	0.0680	2.0925	1.1380	0.9969
	haar	PA	256.8	3.9248	32.492	0.0558	1.7155	0.9330	0.9992
	coif3	PA	299.8	2.5651	8.8463	0.0741	2.2860	1.2446	0.9967
	mexh	PA	248.6^1^	6.6815	54.282	0.0947	2.9212	1.5905	0.9992

Successive divisor									
PA 32.5; IB 20/PA 32.5	haar	CA	263.7^1,2^	238.98	−64.253	3.0612	7.3671	6.4030	0.9993
	coif3	CA	278.8	38.057	3.3329	0.2544	0.6122	0.5321	0.9998

CA 2; PA 32.5/CA 2	sym6	IB	216.8	0.2388	−1.2639	0.0011	0.0243	0.0177	0.9999
	haar	IB	216.6	0.6190	−0.2462	0.0023	0.0538	0.0391	0.9999
	coif3	IB	210.4	0.2345	3.6966	0.0028	0.0649	0.0471	0.9994

PA 32.5; CA 2/PA 32.5	sym6	IB	220.5^1,2^	−29.814	104.61	0.3792	8.7352	6.3452	0.9994
	coif3	IB	242.0	14.208	78.234	0.0638	1.4704	1.0681	0.9999

CA 2; IB 20/CA 2	sym6	PA	251.1	33.526	299.80	0.4087	12.609	6.8653	0.9994
	haar	PA	249.3	−16.153	−140.13	0.1689	5.2124	0.8379	0.9996
	mexh	PA	243.2	10.856	29.469	0.1183	3.6505	1.9875	0.9995

IB 20; CA 2/IB 20	sym6	PA	212.0^1,2^	−165.44	−1490.1	2.5918	79.969	43.539	0.9990
	haar	PA	225.3	−47.847	−313.61	0.3201	9.8771	5.3776	0.9998
	coif3	PA	247.9	9.9188	81.622	0.0758	2.3398	1.2739	0.9998
	mexh	PA	248.6	−10.751	−154.96	0.0988	3.0491	1.6601	0.9997

*Y* = *a* × *C* + *b*, where *C* is the concentration in mg/L and *Y* is in signal's amplitude units; *a*: slope; *b*: intercept; *S*_*a*_: standard error of the slope; *S*_*b*_: standard error of the intercept; *S*_*y.x*_: standard error of the regression; *R*^2^: coefficient of determination. ^*∗*^*Y* = *a* × lnC + *b*. ^1^Wavelengths used for assay (the highest absolute values of the slopes of the developed spectrophotometric methods using the same type of divisor for each compound). ^2^Wavelengths used for the dissolution test (the highest absolute values of the slopes of all the developed spectrophotometric methods for each compound).

**Table 2:** Assay results for the determination of PA, IB, and CA in solid pharmaceutical dosage forms.

% Of label claim (mean ± SD, *n* = 6)									
Method	Bidi-Ipalvic			Ibu-Acetalvic			Glotasic		
	PA	IB	CA	PA	IB	CA	PA	IB	CA
RP-HPLC	100.9 ± 1.9	100.4 ± 2.0	100.5 ± 1.9	101.2 ± 1.9	100.6 ± 2.5	102.5 ± 1.9	101.6 ± 2.5	100.1 ± 2.6	100.9 ± 2.7

*Derivative transform*									
Single divisor	100.0 ± 1.7	99.9 ± 2.1	100.9 ± 1.9	99.9 ± 2.1	98.5 ± 2.6	102.7 ± 2.6	100.9 ± 2.4	98.8 ± 2.7	100.5 ± 1.8
Double divisor	101.3 ± 2.5	99.7 ± 2.4	101.1 ± 1.7	101.1 ± 2.4	101.1 ± 2.4	99.7 ± 2.7	100.6 ± 2.6	100.3 ± 1.9	100.9 ± 1.9
Successive divisor	100.4 ± 1.7	99.6 ± 2.1	100.2 ± 2.3	100.9 ± 2.4	101.7 ± 1.9	101.1 ± 2.5	101.9 ± 2.4	100.6 ± 2.5	99.2 + 2.6

*Fourier transform*									
Double divisor	100.7 ± 2.1	—	—	99.9 ± 2.5	—	—	100.4 ± 2.4	—	—

*Wavelet transform*									
Single divisor	101.3 ± 1.5	100.0 ± 1.4	100.6 ± 1.6	99.6 ± 2.0	101.2 ± 2.5	99.9 ± 2.3	98.8 ± 2.0	100.2 ± 2.1	100.8 ± 2.1
Double divisor	100.1 ± 2.0	100.4 ± 1.8	—	101.9 ± 2.6	99.4 ± 2.3	—	100.6 ± 2.0	100.3 ± 2.2	—
Successive divisor	101.5 ± 2.1	100.7 ± 1.8	100.2 ± 2.4	101.5 ± 2.5	99.9 ± 2.4	99.4 ± 2.5	99.2 ± 2.2	101.2 ± 2.0	101.2 ± 2.5

**Table 3:** Statistical analysis at the significance level 5% for spectrophotometric and liquid chromatographic determination of PA, IB, and CA in three commercial pharmaceutical formulations.

*One-way ANOVA test*					
Source of variation	Compound		Between-groups	Within-groups	Total
Sum of squares	PA	I	13.770	153.55	167.32
		II	30.000	214.00	244.00
		III	47.640	215.65	263.29
	IB	I	6.000	135.10	141.10
		II	46.183	198.40	244.58
		III	18.891	185.80	204.69
	CA	I	9.530	118.60	128.13
		II	63.170	177.25	240.42
		III	15.290	157.80	173.09

Degree of freedom	PA		7	40	47
	IB		6	35	41
	CA		5	30	35

Calculated *F* value	PA	I	0.512		
		II	0.801		
		III	1.262		
	IB	I	0.259		
		II	1.358		
		III	0.593		
	CA	I	0.482		
		II	2.138		
		III	0.581		

Tabulated *F* value	PA		2.249		
	IB		2.372		
	CA		2.534		

*Bartlett test*					
Calculated χ^2^ value	PA	I	1.662		
		II	0.859		
		III	0.615		
	IB	I	1.513		
		II	0.544		
		III	1.042		
	CA	I	1.215		
		II	0.677		
		III	1.334		

Tabulated χ^2^ value (degree of freedom)	PA		14.067 (7)		
	IB		12.592 (6)		
	CA		11.070 (5)		

I, Bidi-Ipalvic; II, Ibu-Acetalvic; III, Glotasic

## Data Availability

No data were used to support this study.

## Supplementary Materials

Supplementary Materials Graphical abstract. New signal-transforming UV spectrophotometric methods for the assay and dissolution test of paracetamol-ibuprofen-caffeine combined tablets and capsules.
