# Supplementary material for: RP-HPLC and UV Spectrophotometric Analysis of Paracetamol, Ibuprofen, and Caffeine in Solid Pharmaceutical Dosage Forms by Derivative, Fourier, and Wavelet Transforms: A Comparison Study
Source: J Anal Methods Chem. 2020 Feb 8;2020:8107571. doi: 10.1155/2020/8107571 (PMC7031732; doi:10.1155/2020/8107571)
Supplement: Supplementary Materials — Graphical abstract. New signal-transforming UV spectrophotometric methods for the assay and dissolution test of paracetamol-ibuprofen-caffeine combined tablets and capsules. [file 8107571.f1.docx]

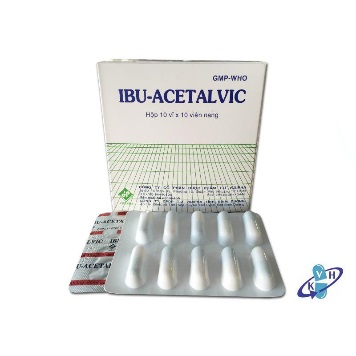

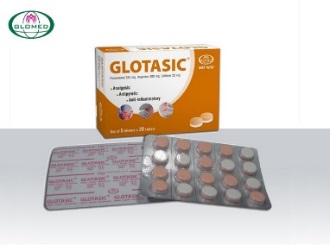

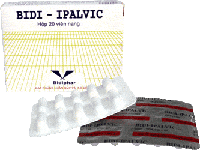


Assay


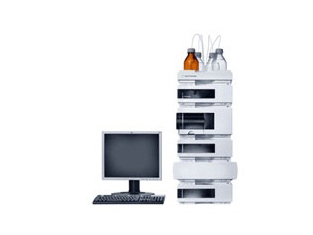


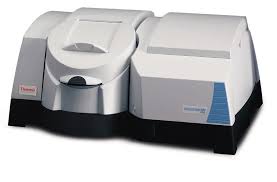


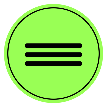


UV spectrophotometry

Derivative, Fourier and wavelet transforms

RP-HPLC


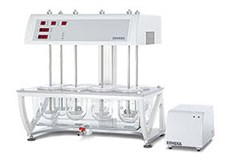


Dissolution test

New signal-transforming UV spectrophotometric methods for the assay and dissolution test of paracetamol-ibuprofen-caffeine combined tablets and capsules
